# Slowing of the Time Course of Acidification Decreases the Acid-Sensing Ion Channel 1a Current Amplitude and Modulates Action Potential Firing in Neurons

**DOI:** 10.3389/fncel.2020.00041

**Published:** 2020-02-28

**Authors:** Omar Alijevic, Olivier Bignucolo, Echrak Hichri, Zhong Peng, Jan P. Kucera, Stephan Kellenberger

**Affiliations:** ^1^Department of Biomedical Sciences, University of Lausanne, Lausanne, Switzerland; ^2^SIB Swiss Institute of Bioinformatics, Lausanne, Switzerland; ^3^Department of Physiology, University of Bern, Bern, Switzerland

**Keywords:** acidification, ASIC, kinetic model, Hodgkin-Huxley model, neuronal signaling, patch-clamp

## Abstract

Acid-sensing ion channels (ASICs) are H^+^-activated neuronal Na^+^ channels. They are involved in fear behavior, learning, neurodegeneration after ischemic stroke and in pain sensation. ASIC activation has so far been studied only with fast pH changes, although the pH changes associated with many roles of ASICs are slow. It is currently not known whether slow pH changes can open ASICs at all. Here, we investigated to which extent slow pH changes can activate ASIC1a channels and induce action potential signaling. To this end, ASIC1a current amplitudes and charge transport in transfected Chinese hamster ovary cells, and ASIC-mediated action potential signaling in cultured cortical neurons were measured in response to defined pH ramps of 1–40 s duration from pH 7.4 to pH 6.6 or 6.0. A kinetic model of the ASIC1a current was developed and integrated into the Hodgkin-Huxley action potential model. Interestingly, whereas the ASIC1a current amplitude decreased with slower pH ramps, action potential firing was higher upon intermediate than fast acidification in cortical neurons. Indeed, fast pH changes (<4 s) induced short action potential bursts, while pH changes of intermediate speed (4–10 s) induced longer bursts. Slower pH changes (>10 s) did in many experiments not generate action potentials. Computer simulations corroborated these observations. We provide here the first description of ASIC function in response to defined slow pH changes. Our study shows that ASIC1a currents, and neuronal activity induced by ASIC1a currents, strongly depend on the speed of pH changes. Importantly, with pH changes that take >10 s to complete, ASIC1a activation is inefficient. Therefore, it is likely that currently unknown modulatory mechanisms allow ASIC activity in situations such as ischemia and inflammation.

## Introduction

Acid-sensing ion channels (ASICs) are Na^+^-conducting channels activated by rapid extracellular acidification (Krishtal and Pidoplichko, 1981; Grunder and Pusch, 2015; Kellenberger and Schild, 2015). ASICs are the targets of several animal toxins (Escoubas et al., 2000; Chen et al., 2005; Baron and Lingueglia, 2015). Animal studies identified roles of ASICs in synaptic signaling which involves rapid pH changes, but also in processes involving slow and sustained pH changes, such as inflammatory pain and neurodegeneration after ischemic stroke (Xiong et al., 2004; Deval et al., 2008; Wemmie et al., 2013).

**Figure 1:**
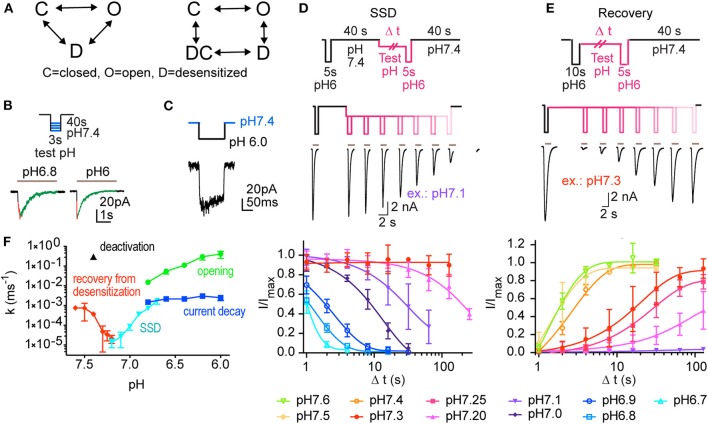
Experiments analyzing the kinetics of ASIC1a. This figure reports the experiments done for creating a kinetic model of ASIC1a function. **(A)** Kinetic models describing ASIC function; left, model containing three functional states (C, closed; O, open; D, desensitized). Right, Hodgkin-Huxley (HH)-type model, in which activation transitions are horizontal, and (de)sensitization transitions are vertical, containing an open, a closed and two desensitized states, D and DC. **(B–E)** Currents were measured by outside-out **(B,C)** or whole-cell **(D,E)** voltage-clamp to −60 mV from CHO cells stably expressing ASIC1a. **(B)** ASICs were activated every 40 s by switching from pH 7.4 to acidic test stimuli for 3 s. The current appearance and decay were each fitted to a single exponential. A schematic protocol and representative current traces are shown. The two traces are from different patches. **(C)** To determine the kinetics of deactivation, the ASICs were activated every 40 s by switching from pH 7.4 to the stimulation pH 6.0 for 120 ms, and then exposed to pH 7.4. **(D)** The kinetics of entry into steady-state desensitization (SSD) were determined with the basic protocol shown on top. Channels were first exposed to pH 6.0 for 5 s (control response), followed by a recovery at pH 7.4 for 40 s. Next, the channels were exposed to a given conditioning pH for different time periods (one duration per sweep), followed by a second switch to pH 6.0 to assess the fraction of the channels having desensitized. Middle panel, pH protocol and representative current traces of an SSD experiment with conditioning pH 7.1. Bottom panel, kinetics of current decay as a function of exposure duration, shown for different conditioning test pH conditions (*n* = 2–11). **(E)** Recovery from desensitization; protocol shown in top panel. Channels were first exposed to pH 6.0 for 10 s, leading to opening and subsequent desensitization. Next, they were exposed to a given conditioning pH for increasing time periods, followed each time by a second exposure to pH 6.0. Middle panel, pH protocol and current traces of a representative experiment with conditioning pH 7.3. Bottom panel, kinetics of current recovery from desensitization, as a function of exposure duration, shown for different recovery test pH (*n* = 3–13). **(F)** Rate constants derived from the different protocols used in B-E, shown as a function of pH. *n* = 3–36 (opening), 11 (deactivation), 2–19 (current decay), 4–8 (SSD kinetics), and 3–11 (recovery from desensitization).

Functional ASICs are homo- or heterotrimeric assemblies of the subunits ASIC1a, −1b, −2a, −2b, and −3. ASIC1a appears to be the most important ASIC subunit in the central nervous system, while ASIC3 contributes importantly to pH sensing in the peripheral nervous system (Wemmie et al., 2013; Kellenberger and Schild, 2015; Sluka and Gregory, 2015). In response to a rapid extracellular acidification, ASICs conduct a transient current that is terminated by desensitization, the entry of the channels in a non-conducting state (Figures 1A,B) (Wemmie et al., 2013; Kellenberger and Schild, 2015). Exposure to pH conditions slightly below physiological values was shown to desensitize the channels without apparent channel opening. This transition has been termed steady-state desensitization (SSD). The entry into the desensitized state is slower (Bonifacio et al., 2014) but occurs at less acidic pH than channel opening. In situations such as strong neuronal signaling, seizures or ischemic stroke, a relatively slow acidification occurs (Chesler, 2003), which may activate ASICs. It seems however also possible that such slow acidification may desensitize ASICs, preventing thereby their opening.

As mentioned above, there is strong evidence for a role of ASICs in processes that are accompanied by slowly developing and sustained acidifications, such as neurodegeneration after cerebral ischemia, and inflammatory pain. ASICs were also shown to contribute to several neurodegenerative diseases (Friese et al., 2007; Arias et al., 2008; Vergo et al., 2011; Huang et al., 2015). A slow acidification occurs also during seizures. ASICs have a dual role in seizures: while the inhibition of ASICs by amiloride reduces seizure probability (Ali et al., 2006; N'Gouemo, 2008; Luszczki et al., 2009), it was also shown that the activation of ASICs can terminate seizures (Ziemann et al., 2008). So far, ASIC currents have almost exclusively been measured in response to fast (<1 s) pH changes, and it is not known how ASICs respond to slow pH changes. In a few studies, a series of small subsequent steps of acidification was applied to cells expressing ASIC3, and it was shown that the peak current was mostly lost in these conditions, while the sustained current was still induced (Yagi et al., 2006; Wang et al., 2013). One study has shown in CNS neurons that a slow pH change (completed in 30–50s) from 7.4 to 6.5 did not induce detectable currents (Bolshakov et al., 2002).

In the present study we focused on ASIC1a and investigated the current response of ASIC1a to slow acidifications, and the induction of action potentials (APs) in neurons by such pH changes. This was done with patch-clamp recordings from Chinese hamster ovary (CHO) cells expressing recombinant ASIC1a channels, with recordings from murine cortical neurons, and in computational models of channel and neuron function. We show that slowing the acidification dramatically decreases ASIC current amplitudes, but increases in turn the duration of the current, leading for intermediately slow acidification (4–10 s duration of pH ramp) to most efficient AP induction in cortical neurons. Slower pH ramps induced only rarely APs.

## Materials and Methods

### Recombinant Expression of ASIC1a in CHO Cells

A Chinese hamster ovary (CHO) cell line stably expressing human ASIC1a [GenBank U78181 (Garcia-Anoveros et al., 1997)] by a vector containing puromycin resistance was used in this study (Poirot et al., 2004). CHO cells had been chosen because of the absence of endogenous H^+^-gated currents in these cells (Poirot et al., 2004; Vukicevic and Kellenberger, 2004). The cells were grown in DMEM/F-12 medium (Thermofisher, Switzerland) supplemented with 3.6% fetal bovine serum (FBS; Thermofisher), 50 units mL^−1^ of penicillin, 50 μg mL^−1^ of streptomycin (Thermofisher) and 10 μg mL^−1^ of puromycin (Fisher Scientific, Reinach, Switzerland).

### Embryonic Mouse Cerebral Cortex Neuron Culture

All animal handling procedures were carried out according to the Swiss federal law on animal welfare and conformed to the European Convention for the Protection of Vertebrate Animals used for Experimental and other Scientific Purposes. The procedures were approved by the committee on animal experimentation of the Canton de Vaud (License VD1750.4). Twenty-one pregnant mice and 126 mouse embryos were used in these experiments to obtain cells for culture; ASIC1a^−/−^ and ASIC2^−/−^ mice (C57BL/6) were provided by Dr. John Wemmie, University of Iowa. Mice used in the experiments were kept in the departmental animal facility at a 12/12 h light dark cycle and had ad libitum access to food and water. Pregnant mice were sacrificed by exposure to CO_2_. The peritoneal cavity was opened to remove the embryos, which were decapitated. Cerebral cortices of WT, ASIC1a^−/−^ or ASIC2^−/−^ fetuses (female and male) of day E14–15 were dissected. They were placed in ice-cold HBSS medium (Thermofisher) complemented with 5 mM HEPES, chopped into small pieces (~1 mm) and incubated at 37°C for 15 min in HBSS medium containing 0.05% Trypsin-EDTA (Thermofisher). The cortical tissues were then washed three times in Neurobasal medium (Thermofisher) containing 10% fetal calf serum (FCS) and dissociated to single cells by gentle trituration using 1 mL blue tip (cut to 0.4 mm diameter) in the Neurobasal/FCS medium containing additionally 1 mg mL^−1^ DNase (DN25, Sigma-Aldrich, Buchs, Switzerland), before centrifugation at 1,000 rpm for 5 min. The neurons were re-suspended in Neurobasal/FCS medium and seeded at 40,000–200,000 cells/dish on 35-mm Petri dishes containing three 15-mm diameter glass coverslips previously coated with poly-L-lysine. After 3 h the medium was replaced by Neurobasal Medium, Electro (Thermofisher) containing B27 serum-free supplement, GlutaMAX supplement (Thermofisher) and gentamicin (10 μg ml^−1^ final concentration; Thermofisher). Neuronal cultures were maintained in a humidified incubator (37°C, 5% CO_2_) and every 2–3 days half of the medium was replaced with fresh plating medium. Patch-clamp experiments of cortical neurons were performed in a Warner Instruments RC-42LP measuring chamber (Warner Harvard Bioscience, Les Ulis, France) after at least 14 days after seeding.

### Electrophysiological Measurements

Electrophysiological measurements were carried out at 20–25°C using the whole-cell or outside-out configuration of the patch-clamp technique in voltage- and current-clamp mode with an EPC10 patch-clamp amplifier (HEKA Elektronik-Harvard Bioscience, Lambrecht, Germany). Data were acquired with Patchmaster software and analysis of the currents, potentials, and time constants was carried out with Fitmaster (HEKA Elektronik-Harvard Bioscience). The sampling interval and the low-pass filtering were set to 1 ms and to 3 kHz, respectively (whole-cell voltage-clamp), 0.02 ms and 5 kHz (outside-out patches; current-clamp experiments of Figure 8), and 1 or 0.2 ms and 5 kHz (other current-clamp experiments).

Recording pipettes were made of borosilicate glass (thin-wall capillaries for whole-cell recordings, thick-wall capillaries for outside-out patches), World Precision Instruments, Hertfordshire, UK) with a Narishige PC-10 puller (Narishige, London, UK) and had resistances between 2 and 4 MΩ (9–11 MΩ for outside-out patches) when filled with the pipette solution. In voltage-clamp experiments the series resistance compensation was set to 70–90% and the holding potential to −60 mV or as indicated. The extracellular Tyrode solution contained, in mM, 140 NaCl, 4 KCl, 2 CaCl_2_, 1 MgCl_2_, 10 HEPES, 10 MES, 10 glucose; pH was adjusted to the desired value using NaOH or HCl. The pipette solution used for voltage-clamp experiments contained (in mM) 90 K gluconate, 10 NaCl, 10 KCl, 1 MgCl_2_, 60 HEPES, 10 EGTA; pH was adjusted to pH 7.3 with KOH. The pipette solution for current-clamp experiments contained (in mM) 90 K gluconate, 10 NaCl, 10 KCl, 3 MgCl_2_, 2 ATP-N_2_, 0.3 GTP-Li^+^, 60 HEPES, 10 EGTA; pH was adjusted to pH 7.3 with KOH. The pH of the solutions was controlled on the day of the experiment and adjusted if necessary.

For whole-cell experiments, rapid solution exchange was achieved using computer-controlled electrovalves (cF-8VS) and the MPRE8 perfusion head (Cell MicroControls, Norfolk, VA). pH ramps were generated using a pair of programmable pumps with 60 ml syringes (Aladdin syringe pump, World Precision Instruments), whose output came together in a perfusion head. The hyperterminal program (Windows Operating System) was used for commanding the two syringe pumps. The total output rate of the two syringe pumps together was maintained constant at 100 ml h^−1^. For outside-out voltage-clamp experiments, solution exchange was achieved with an ultrarapid piezo-driven system (MXPZT- 300L; Siskiyou, Grants Pass, OR, USA) and a perfusion head made of a two-barrel borosilicate theta tube (Warner Instruments: TG200-4) under a continuous flow at 10 ml h^−1^ controlled by two syringe pumps (Aladdin syringe pump, World Precision Instruments: AL-2000). The outer tip diameter of the theta tubing was adjusted to ~100 μm. For display, data obtained from outside-out patches were digitally filtered at 100 Hz. In current-clamp experiments, baseline current was injected to obtain a membrane potential close to −60 mV.

### Cell Surface pH Imaging

pH imaging experiments were performed using an inverted fluorescence microscope (Zeiss Axio Observer, AX10, Feldbach, Switzerland) with a 63x objective (Plan-Apochromat) and a CoolSnap HQ2 camera (Photometrics, Tucson, USA). The time resolution of the imaging part was ~ 440 ms. Images were recorded using the Metafluor software (Molecular Devices, Sunnyvale, USA). Briefly, cells were grown in Fluorodish cell culture dishes (World Precision Instruments) or on 15-mm diameter glass coverslips (VWR, Dietikon, Switzerland) and incubated prior to the experiment in Tyrode solution containing 10 μM of 5(6)-FAM SE [5-(and-6)-Carboxyfluorescein, succinimidyl ester], mixed isomers (BIOTIUM, Chemie Brunschwig, Basel, Switzerland), a ratiometric dye able to sense pH changes (excitation 460/488 nm, emission 520 nm) for 15 min in an incubator (37°C, 5% CO_2_). The amine-reactive succinimidyl ester form of the dye binds exclusively amine groups on cell surface proteins. The baseline of the 5(6)-FAM SE ratio measured during the perfusion of the Tyrode solution showed a certain degree of photobleaching. We corrected for the photobleaching using Origin PRO software (OriginLab Corp, Northampton, USA). After baseline correction, the 90–10% fall time of the fluorescence signal was determined to classify the time course of the pH changes.

### Kinetic Model of ASICs

#### Attempts at Developing a Markovian Model

In a first attempt, an ASIC1a model was generated based on the 3-state kinetic scheme [(closed (C), open (O), and desensitized (D), Figure 1A, left]. Five of the rate constants (k_CO_, k_OC_, k_OD_, k_CD_, and k_DC_) were obtained by fitting exponential or sigmoid functions of pH to the rates measured experimentally (Figure 1F). The sixth rate constant (k_DO_) was calculated based on microscopic reversibility (Sakmann and Neher, 1995). However, when subjected to pH protocols corresponding to the experiments, the model did not describe ASIC1a behavior as well as the model developed using a Hodgkin-Huxley formalism that will be presented below. In particular, it did not replicate sufficiently well the rates measured experimentally. This observation is not necessarily surprising because experimental rate constants are not independent parameters and rate constants in kinetic models cannot always be inferred from them (Milescu et al., 2005).

In a second step, we modeled the rate constants as functions of the form k = a·[H^+^]^b^, which assumes ligand-binding kinetics to the channel with H^+^ as ligand. This led to 10 free parameters to be estimated (6 rates with 2 parameters each, minus 2 parameters being constrained by microscopic reversibility). Estimation was conducted by non-linear minimization of a cost function determined by the squared residuals to the experimental data. The parameters could however not be identified, as different initial guesses converged to different parameter sets. However, the rates k_CD_ and k_DC_ were always orders of magnitude lower than the other rates. We therefore attempted to model ASIC1a function using a linear C⇆O⇆D model (8 free parameters, 4 rates with 2 parameters each). Although different initial guesses converged to a unique solution, the model was still not able to satisfactorily replicate the experimental results. In particular, the overall shape of the experimental rates represented as a function of pH (Figure 1F) could not be reproduced.

These observations suggest that more states should be incorporated into the kinetic model and/or that the rate constants must be more elaborate functions of pH. Both scenarios imply increasing the number of parameters, which in both cases precludes their identifiability (Fink and Noble, 2009) based on the available experimental data.

#### Modeling Based on a Hodgkin-Huxley Approach

For these reasons, we decided to use a more empirical approach based on the work of Hodgkin and Huxley (Hodgkin and Huxley, 1952). The data in Figure 1 clearly demonstrate two opposing processes (activation and desensitization) as well as the presence of two characteristic pH-dependent rate constants differing by 1–2 orders of magnitude (Figure 1F). Therefore, I_ASIC1a_ was represented as

$$\begin{array}{l} {\text{I}}_{ASIC1a}=g_{\text{ASIC}1\text{a}}\cdot a\cdot s\cdot \left({\text{V}}_{\text{m}}-{\text{E}}_{\text{ASIC}1\text{a}}\right) \end{array}$$

with a rapid activation gate *a* and a slow sensitization/desensitization gate *s*. The dynamic behavior of *a* and *s* are governed by their steady-state values *a*_∞_ and *s*_∞_ as well as by their rates *r*_*a*_ and *r*_*s*_, respectively.

**Figure 2:**
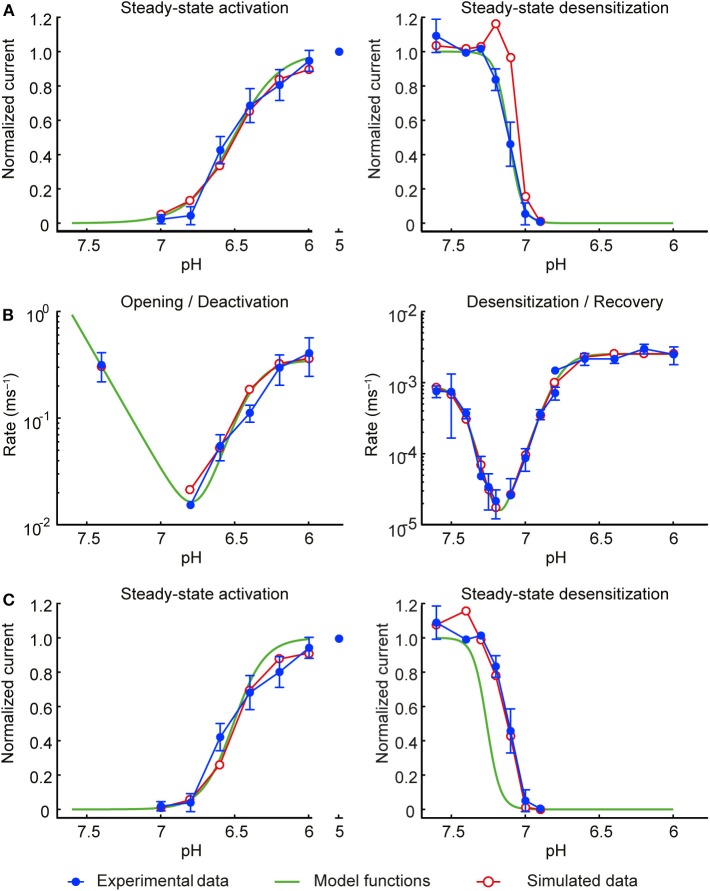
Development of the model of the ASIC1a current. Experimental data are shown in blue, the initial functions *a*_∞_ and *s*_∞_ fit to the experimental data are shown in green and the corresponding simulated data in red. Currents were measured from CHO cells stably expressing ASIC1a, in the whole-cell mode (**A,C**; **B**, right panel, desensitization from the closed state and recovery from desensitization) or from excised outside-out patches [**B**, left panel (opening and deactivation) and right panel, desensitization from the open state]. **(A)** Steady-state activation (left) and steady-state desensitization (SSD, right). For measuring steady-state activation curves, cells were exposed to the conditioning pH 7.4, and the solution was changed every 50 s for 5 s to an acidic test pH to activate ASICs, *n* = 8–10. To determine the SSD pH dependence, channels were activated by a first exposure to pH 6 for 8 s (I_1_), which was followed by a 40-s exposure to pH 7.4, a 60-s exposure to the test conditioning pH and a 8-s exposure to pH 6 (I_2_). After this, channels were exposed during 42 s to pH 7.4, and subsequently a new cycle, with a different test conditioning pH was started. The I_2_/I_1_ ratio was then plotted as a function of the conditioning pH, *n* = 4–9. **(B)** Rates of opening and deactivation (left) and desensitization and recovery from desensitization (right). Experimental data are as in Figure 1F; protocols as described in the legend of Figure 1. **(C)** Same as in **(A)**, but using the modified functions *a*_∞_ and *s*_∞_ of the final model. In all simulations, the model was subjected to the same pH protocols and its output to the same analyses as in the experiments.

To find the steady-state functions *a*_∞_ and *s*_∞_, data from steady-state activation and steady-state desensitization protocols were fitted (least squares) with functions of pH (green curves in Figure 2) of the form:

$$\begin{array}{l} f\left(\text{pH}\right)=\frac{1}{1+k\cdot 10^{h\left(\text{pH}-7\right)}}\; \end{array}$$

with *k* = 23.67 and *h* = 2.797 for gate *a*, and *k* = 11.18 and *h* = −9.448 for gate *s*, where the *k* controls the position of the steady-state activation and desensitization curves along the pH axis and *h* is a slope factor (corresponding to a Hill coefficient). pH−7 was used instead of pH to describe deviation from acid-base neutrality. The pH at which *f* (pH) = ½ (midpoint) is at pH = 7–(1/h)·log_10_(k).

The rapid and slow rate constants *r*_*a*_ and *r*_*s*_ were obtained by fitting the natural logarithms of the measured rate constants rather than the rate constants themselves (or the corresponding time constants). This approach offers the advantage to not give excessive weight to large values and insufficient weight to small values. The logarithm ln(*r*) of the measured rates (in ms^−1^) were fitted individually for the fast and slow processes (see Figure 1F) using functions (green curves in Figure 2B) of the form

$$\begin{array}{l} \text{ln}\left(r\right)=\text{ln}\left(\frac{a_{1}}{1+e^{b_{1}\left(\text{pH}-c_{1}\right)}}+\frac{a_{2}}{1+e^{b_{2}\left(\text{pH}-c_{2}\right)}}\right) \end{array}$$

with *a*_1_ = 0.3487, *b*_1_ = 10.14, *c*_1_ = 6.392, *a*_2_ = 234.3, *b*_2_ = −5.553 and *c*_2_ = 8.595 for gate *a* (giving *r*_*a*_) and with *a*_1_ = 0.002531, *b*_1_ = 13.94, *c*_1_ = 6.769, *a*_2_ = 0.0008942, *b*_2_ = −18.39 and *c*_2_ = 7.436 for gate *s* (giving *r*_*s*_).

This initial model was then subjected to exactly the same pH protocols as in the experiments (same sequence of pH steps and the same intervals between the successive sweeps). To account for the non-instantaneous changes of pH inherent to the experimental system, the pH changes were modeled as ramps lasting 1 ms (excised outside-out patches) or 200 ms (whole cells). The simulated I_ASIC1a_ was then analyzed in the same manner as in the experiments to compute steady-state activation and desensitization curves and rate constants, as illustrated by the red symbols in Figures 2A,B. While steady-state activation and the rate constants were well-reproduced, a clear discrepancy was apparent for the steady-state desensitization curve. This discrepancy is due to the fact that in the steady-state desensitization protocol, the duration of the conditioning pH step was 1 min, which, in view of the very slow rates reported in Figure 1F (near 10^−5^ ms^−1^), is too short to obtain complete desensitization. This explains the shift of the reconstructed curve (red symbols in the right panel of Figure 2A) toward lower pH values upon reconstructing the experimental protocol. Therefore, to compensate for this, the function *s*_∞_ was shifted toward higher pH values by 0.15 pH units (green curve in the right panel of Figure 2C).

**Figure 3:**
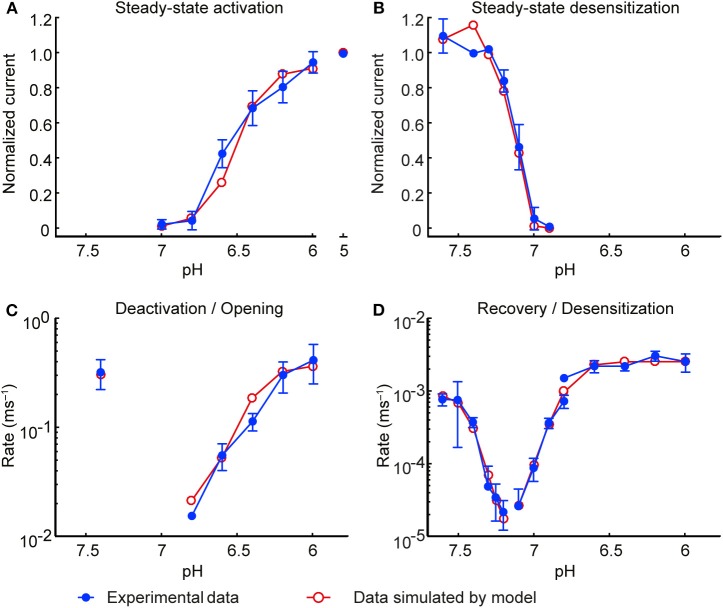
Model of the ASIC1a current: simulations vs. experiments. Experimental data are shown in blue and model results in red. Currents were measured from CHO cells stably expressing ASIC1a, in the whole-cell mode (**A,B**; **D** except for desensitization from the open state) or from excised outside-out patches (**C**; **D**, desensitization from the open state). **(A)** Steady-state activation. **(B)** Steady-state desensitization (SSD). Experimental data in A and B are the same as in Figures 2A,C; the corresponding protocols are described in the legend of Figure 2. **(C)** Rate of opening and deactivation. **(D)** Rate of desensitization and recovery from desensitization. Experimental data in **(C,D)** are the same as in Figure 1F. In all simulations, the model was subjected to the same pH protocols and its output to the same analyses in the experiments.

The slight overlap of the functions *a*_∞_ and *s*_∞_ led to a window current (product *a*_∞_·*s*_∞_) that overestimated the peak current registered experimentally during slow pH ramp protocols. To decrease this window current, the steepness of the steady-state activation function *a*_∞_ was increased by a factor 1.48. This value corresponds to the upper bound of the 95% confidence interval of the fitted slope factor *h*. Figure 2C (left panel) shows that the modified *a*_∞_ still represented well the values determined experimentally. The refined model was then validated by subjecting it once more to the experimental protocols and analyses, resulting in good agreement with the experimental data (Figures 2C, 3). The functions *a*_∞_, *s*_∞_, *r*_*a*_, and *r*_*s*_ of this final model are provided in Table 1.

**Table 1:** Model gating functions and parameters.

**Gate**	**Steady-state**	**Rate**
**I**_**ASIC1a**_ **model**		
*a*	$a_{\infty }=\frac{1}{1+23.67^{1.48}\cdot 10^{1.48\cdot 2.797\left(pH-7\right)}}$	$r_{a}=\frac{0.3487}{1+e^{10.14\left(pH-6.392\right)}}+\frac{234.3}{1+e^{-5.553\left(pH-8.595\right)}}$
*s*	$s_{\infty }=\frac{1}{1+11.18\cdot 10^{-9.448\left(pH-7-0.15\right)}}$	$r_{s}=\frac{0.002531}{1+e^{13.94\left(pH-6.769\right)}}+\frac{0.0008942}{1+e^{-18.39\left(pH-7.436\right)}}$
**Gate**	**Opening rate**	**Closing rate**
**Hodgkin-Huxley action potential model**		
*m*	$\alpha_{m}=\frac{2.5-0.1\left(V_{m}+60\right)}{e^{2.5-0.1\left(V_{m}+60\right)}-1}$	$\beta_{m}=4e^{-\left(V_{m}+60\right)/18}$
*h*	$\alpha_{h}=0.007e^{-\left(V_{m}+60\right)/20}$	$\beta_{h}=\frac{0.1}{{1+e}^{3-0.1\left(V_{m}+60\right)}}$
*n*	$\alpha_{n}=\frac{0.1-0.01\left(V_{m}+60\right)}{e^{1-0.1\left(V_{m}+60\right)}-1}$	$\beta_{n}=0.125e^{-\left(V_{m}+60\right)/80}$

*The Hodgkin-Huxley action potential model is based on Hodgkin and Huxley (1952). a is the activation gate and s is the sensitization/desensitization gate of the I_ASIC1a_ model, m is the activation gate and h is the inactivation gate of the Hodgkin-Huxley Na^+^ current model, n is the activation gate of the Hodgkin-Huxley K^+^ current model. Rates are in ms^−1^, membrane potential (V_m_) in mV. The maximal conductance parameters are the maximal conductance of ASIC1a (g_ASIC1a_), sodium (g_Na_) and potassium (g_K_) channels, and leak current (g_L_). Some of these parameters were adapted in our model with regard to the original parameters (in mS cm^−2^) that are shown in parentheses: g_Na_: 135 (120), g_K_: 45 (36), g_L_: 0.5 (0.3); g_ASIC1a_ was set to 10 mS cm^−2^*.

#### Incorporation of I_ASIC1a_ Into the Hodgkin-Huxley Model of the Nerve Action Potential

To investigate the effects of slow pH changes on the AP generation in neurons, the I_ASIC1a_ model was incorporated into the Hodgkin-Huxley model (Hodgkin and Huxley, 1952):

$$\begin{array}{l} c_{m}\frac{\text{d}{\text{V}}_{\text{m}}}{\text{dt}}=-(g_{\text{Na}}m^{3}h\left({\text{V}}_{\text{m}}-{\text{E}}_{\text{Na}}\right)+g_{\text{K}}n^{4}\left({\text{V}}_{\text{m}}-{\text{E}}_{\text{K}}\right)+\\ \;g_{L}\left({\text{V}}_{\text{m}}-{\text{E}}_{\text{L}}\right)+{\text{I}}_{\text{ASIC}1\text{a}}+{\text{I}}_{\text{stim}}) \end{array}$$

where *c*_*m*_ = 1 μF cm^−2^ is the membrane capacitance, V_m_ is the membrane potential, *g*_Na_·m^3^h(V_m_-E_Na_) is the Na^+^ current (with three activation gates *m* and one inactivation gate *h*), *g*_K_·n^4^(V_m_−E_K_) is the K^+^ current (with four activation gates *n*), *g*_L_·(V_m_−E_L_) is the leak current and I_stim_ is the externally applied stimulation current. The maximal conductances of the Na^+^, K^+^ and leak currents were *g*_Na_ = 135 mS cm^−2^, *g*_K_ = 45 mS cm^−2^, *g*_L_ = 0.5 mS cm^−2^, respectively, and E_Na_ = +55 mV, E_K_ = −72 mV and E_L_ = −49.387 mV are their corresponding Nernst/reversal potentials. Resting V_m_ was −60 mV. Unless specified otherwise, the maximal conductance *g*_ASIC1a_ of I_ASIC1a_ was set to 10 mS cm^−2^ and its reversal potential E_ASIC1a_ to +50 mV.

The gating variables of the Na^+^ and K^+^ currents were governed by differential equations of the form

$$\begin{array}{l} \frac{\text{d}x}{\text{d}t}=\alpha_{x}\left(1-x\right)-\beta_{x}x\; \end{array}$$

where *x* stands for any of the gating variables (*m, h* or *n*), α_*x*_ is the corresponding opening rate and β_*x*_ the corresponding closing rate. α_*x*_ and β_*x*_ are functions of *V*_*m*_ and are given in Table 1.

To explore how different voltage dependencies and kinetics of Na^+^ current activation and inactivation influence the manner I_ASIC1a_ elicits action potentials, the functions α_x_ and β_x_ were either shifted along the voltage axis by an amount s_x_ or scaled by a common factor f_x_ (this accelerates or decelerates the kinetics without altering the voltage dependence). Shift along the voltage axis was done by substituting V_m_ by V_m_ + s_x_ in the corresponding functions listed in Table 1, and scaling was done by multiplication by f_x_. These analyses were conducted for both gates *m* (Na^+^ current activation) and *h* (Na^+^ current inactivation).

#### Instantaneous pH Changes and pH Ramps

**Figure 4:**
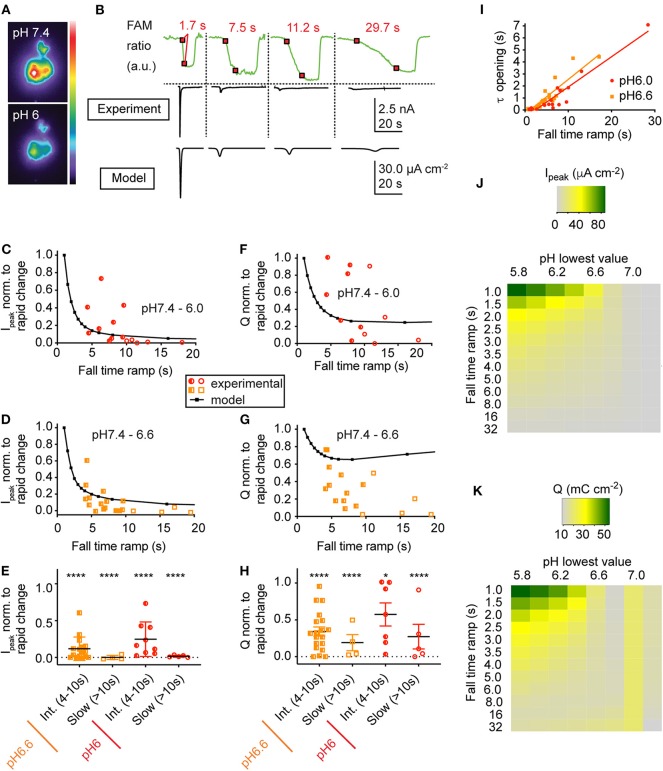
Slowing of the pH change reduces the current amplitude and the transported charge. **(A)** Pseudocolor image by the pH-sensitive fluorescent dye 5(6)-FAM of the same cell at pH 7.4 and at pH 6.0. **(B)** Fluorescence intensities (green traces, upper panel) as a function of time in response to pH ramps of different speed from pH 7.4 to pH 6.0. The 10–90% fall-time (FT) of the fluorescence change corresponds to the interval between the two red squares (Materials and Methods). Each fluorescence trace (top panel) was measured simultaneously with the corresponding ASIC1a current trace (middle panel). Bottom panel, traces created by the kinetic model in response to the above pH ramps. The experimental data in **(B–I)** are from whole-cell patch-clamp at −60 mV. **(B–H)** Current and transported charge values are normalized to the corresponding response measured in the same cell to a rapid pH change (ramp FT < 4 s). **(C,D)** ASIC1a peak current amplitudes in response to pH 7.4–6.0 ramps **(C)** or pH 7.4–6.6 ramps **(D)** of different duration, shown as function of pH ramp FT (*n* = 14–20). The model prediction is indicated with black symbols and the connecting line. **(E)** Normalized peak current amplitudes for ramp durations classified into intermediate (ramp FT 4–10 s) and slow (ramp FT > 10 s; *n* = 4–16). ****p < 0.0001, different from rapid change (ANOVA, Tukey *post-hoc* test). **(F,G)** Transported charge (Q), the current integral over time, is indicated for the same protocols as shown in **(C,D)** (*n* = 12–17). The model prediction is indicated with black symbols and the connecting line. **(H)** Normalized transported charge for ramp durations classified as in **E** (*n* = 4–16). *p < 0.05; ****p < 0.0001, different from rapid change (ANOVA, Tukey *post-hoc* test). **(I)** Time constant of ASIC current appearance in pH 7.4–6.0 (red symbols) and pH 7.4–6.6 ramps (orange), plotted as a function of the pH ramp FT. The *r*^2^ values were 0.906 (*p* < 0.001, pH 7.4–6.0 ramps) and 0.846 (*p* < 0.001, pH 7.4–6.0 ramps), *n* = 21–29. **(J,K)** Heat map of the simulated peak ASIC1a current **(J)** or charge transport **(K)** as a function of the pH ramp lowest value (pH) and duration (FT) in seconds. Shown are the amplitude of the maximal current (I_peak_, μA cm^−2^) and the transported charge (Q, mC cm^−2^) according to the scaling bar.

For modeling instantaneous pH changes we considered an initial pH at time 0 which was switched at time > t_switch_ to another pH and was held constant onwards (pH step from the initial pH to the final pH). For modeling the effects of slow pH changes (pH ramps), the pH was first held for 5,000 ms at the initial pH (normally 7.4). The pH was then changed linearly with time as pH(t) = pH(0)−q*t*, q being the rate of change of the pH, determined by the final pH and the duration of the ramp. The pH was then maintained at the final pH. To simulate pH ramps similar to those used in experiments in which two solutions were mixed (Figure 4B), we used a sigmoid-shaped function consisting of piecewise quadratic, linear and constant functions defined as follows:

$$\begin{array}{l} \begin{array}{cccc} \text{pH}\left(\text{t}\right)={\text{pH}}_{\text{i}} & \text{for} & \; & \text{t}\leq {\text{t}}_{\text{i}}\\ {\text{a}}_{1}\cdot {\text{t}}^{2}+{\text{b}}_{1}\cdot \text{t}+{\text{pH}}_{\text{i}} & \text{for} & {\text{t}}_{\text{i}} & <\text{t}\leq {\text{t}}_{\text{i}}+\text{rt}\cdot 0.5\\ {\text{a}}_{2}\cdot \text{t}+{\text{b}}_{2} & \text{for} & {\text{t}}_{\text{i}}+\text{rt}\cdot 0.5 & <\text{t}\leq {\text{t}}_{\text{i}}+\text{rt}\cdot 1.5\\ {\text{a}}_{3}\cdot {\text{t}}^{2}+{\text{b}}_{3}\cdot \text{t}+\text{p}{\text{H}}_{\text{f}} & \text{for} & {\text{t}}_{\text{i}}+\text{rt}\cdot 1.5 & <\text{t}\leq {\text{t}}_{\text{i}}+\text{rt}\cdot 2.0\\ \text{p}{\text{H}}_{\text{f}} & \text{for} & {\text{t}}_{\text{i}}+\text{rt}\cdot 2.0 & <\text{t} \end{array} \end{array}$$

where pH_i_ and pH_f_ represent the initial and final pH values, and t_i_ and rt represent the start and rise times of the pH drop, respectively. The coefficients a_1_, b_1_, a_2_, b_2_, a_3_, and b_3_ are parameters depending on the pH drop amplitude and rise time. These coefficients were determined such that pH(t) and its 1st derivative (dpH(t)/dt) are both continuous at the four transitions between the piecewise functions (i.e., at t = t_i_, t = t_i_ + rt·0.5, t = t_i_ + rt·1.5 and t = t_i_ + rt·2.0).

#### Numerical Methods

In simulations of I_ASIC1a_ in the voltage-clamp mode, state occupancies or gating variables were integrated using the forward Euler method with a constant time step of 0.1 or 0.01 ms. In simulations of nerve activity (current-clamp mode), this time step was set to 0.01 ms to integrate the gating variables and membrane potential. In all simulations, initial conditions for the gates were obtained from the equilibrium at the initial pH and resting membrane potential. All simulations were run using MATLAB (The MathWorks, Natick, MA, USA).

### Analysis and Statistics

The pH activation curves were fitted using the Hill equation:

$$\begin{array}{l} I\;=\;\frac{I_{max}}{{1+\left(\frac{10^{-pH50}}{10^{-pH}}\right)}^{nH}}\;; \end{array}$$

where *Imax* is the maximal current amplitude, *pH*_50_ is the pH at which half of the maximal current is measured, and *nH* is the Hill coefficient. Steady-state desensitization (SSD) curves were fitted by an analogous equation.

Transition kinetics were fitted to mono-exponential functions. The number of APs in current-clamp experiments was determined with FitMaster (HEKA Elektronik-Harvard Bioscience), measured over the same duration in pH ramps of different duration.

The maximal dV_m_/dt in phase-plane plots was determined as the maximal value in the plot. The threshold was determined by the intersection of two tangents that were fitted to the initial flat part and to the subsequent steep part of the dV_m_/dt vs. V_m_ plots, respectively.

The normality of the data distribution was determined by using the D'Agostino & Pearson normality test. For normally distribution data we used one-way ANOVA followed by Tukey *post-hoc* test. For not normally distributed data we used the Kruskal-Wallis followed by Dunn's *post-hoc* test. *P*-values of < 0.05 were considered significant. Statistical tests were carried out with Graphpad Prism8 (San Diego, USA). Regression calculations were performed with the statistical software R (http://www.R-project.org/). Data are presented as individual data points or as mean ± SD. The datasets of the experimental data are provided in the Supplementary Table 1.

## Results

### Measurement of ASIC1a Kinetics for Establishing a Kinetic Model

To measure the kinetics of transitions between the functional states of ASIC1a for the establishment of a kinetic model, several types of pH protocols were applied in patch-clamp experiments to CHO cells stably expressing ASIC1a (Materials and Methods). The kinetics of rapid transitions [k > 2 s^−1^; for opening [C-O in the COD model, Figure 1A], open channel desensitization (O-D) and deactivation [O-C]] were measured from outside-out patches. Slower processes [steady-state desensitization (SSD), [C-D], and recovery from desensitization [D-C]], were measured in the whole-cell configuration. Representative traces show activation of ASIC1a by pH 6.8 and 6.0 from a conditioning pH of 7.4 with fast solution changes to excised outside-out patches (Figure 1B). Such experiments allowed measuring the kinetics of channel opening [C-O] and of current decay [O-D].

To determine the kinetics of deactivation, the pH was changed from 7.4 to 6.0 for 120 ms, and was then changed back to pH 7.4 before substantial desensitization had occurred (Figure 1C). This showed that the deactivation time course is of the same order as the opening time course. To measure the kinetics of SSD, the protocol illustrated in Figure 1D was applied, in which channels were exposed to a test conditioning pH (7.1 in the example in Figure 1D) for increasing durations, to assess the fraction of channels that desensitize during this exposure. Such protocols were applied with different conditioning pH values, and the results are shown in Figure 1D (bottom panel), indicating as expected that the desensitization kinetics are faster, the more acidic the conditioning pH is. To measure the kinetics of the recovery from desensitization at a given recovery pH, a control response to pH 6.0 was recorded, which desensitized all channels. Channels were then exposed to the recovery pH (7.3 for the experiment shown in Figure 1E) for increasing durations, before a second pH 6.0 stimulation was used to assess the fraction of channels that had transited from the desensitized to the closed state. The results of such protocols applied at diverse recovery pH values are shown in Figure 1E (bottom panel) and indicate that recovery is faster at more alkaline pH. The rate constants of these different transitions are plotted in Figure 1F as a function of the pH. This figure shows rapid transitions (opening and deactivation) as well as slow transitions (current decay, SSD, recovery from desensitization). The kinetics of the current decay show no obvious pH dependence, whereas the other transitions are pH-dependent. Although we provide measurements of deactivation only at pH 7.4, this transition was previously shown to decelerate with acidic pH (MacLean and Jayaraman, 2017).

### Generation of a Kinetic Model of ASIC1a

Two strategies were tested to generate a kinetic model of the ASIC1a current (I_ASIC1a_), a 3-state Markovian COD model and a model based on the framework of Hodgkin and Huxley (HH; Figure 1A). Since the HH-based model fitted the experimental data better than the COD model, as explained in Materials and Methods, only the HH-based model is described here. The HH-based model contains a rapid activation (opening/closing) gate *a* and a slow sensitization/desensitization gate *s*. Gate *a* controls the transitions between states C and O as well as between states DC and D (Figure 1A, right). Conversely, gate *s* controls the transitions between states D and O as well as between states DC and C. I_ASIC1a_ was represented as

$$\begin{array}{l} {\text{I}}_{\text{ASIC}1\text{a}}={\text{g}}_{\text{ASIC}1\text{a}}\cdot \text{a}\cdot \text{s}\cdot \left({\text{V}}_{\text{m}}-{\text{E}}_{\text{ASIC}1\text{a}}\right)\; \end{array}$$

where *g*_*ASIC*1*a*_ is the maximal conductance of *I*_*ASIC*1*a*_, *V*_*m*_ is the membrane potential, *E*_*ASIC*1*a*_ is the reversal potential of *I*_*ASIC*1*a*_, *a* is the activation variable and *s* is the sensitization/desensitization variable. The time-dependent behavior of the gating variables *a* and *s* was governed by

$$\begin{array}{l} \frac{\text{d}a}{\text{d}t}=r_{a}\cdot \left(a_{\infty }-a\right)\; \end{array}$$

and

$$\begin{array}{l} \frac{\text{d}s}{\text{d}t}=r_{s}\cdot \left(s_{\infty }-s\right)\; \end{array}$$

where *a*_∞_ and *s*_∞_ are the steady-state values and *r*_*a*_ and *r*_*s*_ are the rates of the gates *a* and *s*, respectively. These steady-state values and rates are functions of pH. These functions as well as their determination and optimization are presented in “Materials and Methods.”

The model was validated by subjecting it to exactly the same pH protocols as in the experiments (including the same sequence of pH steps and the same intervals between the successive sweeps). Moreover, to account for the non-instantaneous changes of pH inherent to the experimental system, the pH changes were not modeled as steps but as ramps lasting 1 ms (for excised outside-out patches) or 200 ms (for whole-cell measurements). The simulated I_ASIC1a_ was then analyzed in the same manner as the experimentally recorded I_ASIC1a_ to compute steady-state activation and desensitization curves (Figures 3A,B, respectively) and rate constants (Figures 3C,D). Figure 3 shows that the values obtained with the I_ASIC1a_ model are in close agreement with the values obtained from the experiments.

### Slowing of the pH Change Leads to Decreased ASIC1a Current Amplitudes

In order to measure the ASIC response to slow pH changes, a pH ramp was applied to ASIC1a-expressing CHO cells under patch-clamp, by using two computer-controlled syringe pumps holding solutions of pH 7.4 and an acidic pH (6.6 or 6.0), whose outputs came together in a perfusion head placed near the cell under study (Materials and Methods). Prior to the experiment, the cells were labeled with a membrane-impermeable, pH-sensitive fluorophore (Materials and Methods). This allowed simultaneous recording of ASIC currents and pH changes at the cell surface (Figures 4A,B). ASIC1a currents were measured in response to pH ramps from pH 7.4 to either pH 6.6 or pH 6.0 at different ramp speeds. The middle panel in Figure 4B shows representative current traces obtained with the different pH 7.4–6.0 ramps whose speed is indicated by the fluorescence changes shown in the top panel. Comparison with the current traces generated by the HH-based ASIC1a model in response to the same pH ramps (Figure 4B, bottom panel) indicates that the kinetic model produces current traces that are similar to the experimental data. From each cell, ASIC current was recorded in response to one fast [fall time (FT) <4 s] and one or several slower pH ramps (“Intermediately slow,” FT 4–10 s; or “slow,” FT >10 s). The current amplitudes produced by ramps with FT > 4 s were normalized to the current amplitude induced by a fast pH change (FT < 4 s) in the same cell and are plotted for pH 7.4–6.0 ramps in Figure 4C. Corresponding results obtained with pH 7.4–6.6 ramps are shown in Figure 4D. Slowing of the pH ramps led to significantly smaller current amplitudes than fast ramps (Figure 4E). This decrease in peak current amplitudes was reproduced by the kinetic model of ASIC1a (solid black lines in Figures 4C,D).

The transported charge, determined as the integral of the current over time, decreased with slowing of the pH change in pH 7.4–6.6 ramps, while such a correlation was less obvious with pH 7.4–6.0 ramps (Figures 4F,G). The charge transport was however in both, pH 7.4–6.6 and pH 7.4–6.0 ramps significantly lowered for ramp FT > 4 s (Figure 4H). The decrease in charge transport appeared less pronounced than the decrease in I_peak_ (Figures 4E,H). This is likely due to the apparent deceleration of the kinetics of current appearance with slower pH ramps (Figure 4I), which allowed a considerable charge entry into the cell, even when the I_peak_ was small. While the model predicted the decay of charge transport in the pH 7.4–6.0 ramp reasonably well (Figure 4F), it predicted for the pH 7.4–6.6 ramps higher values than those found in the experiments (Figure 4G).

The kinetic model was then used to predict ASIC1a current and charge transport for a range of ramp end pH values (7.2–5.8; i.e., pH ramps starting at pH 7.4 and leading to the indicated value) and of speeds of the pH ramp (1–32 s, Figures 4J,K). This showed that for both, I_peak_ and Q, the model predicts a decrease with more alkaline ramp end pH and with the slowing of the pH ramp. This decrease was steeper for the currents than the charge transport. To induce ion current or charge transport, an acidification to ≤pH 6.7 was necessary in the model. This is close to pH 6.8, which induces in experimental settings a small current (Vukicevic and Kellenberger, 2004). The model prediction of charge transport in the range of pH 7.0–7.15 was not observed in experiments. In conclusion of these computational and experimental approaches, pH ramps induced ASIC1a currents only if they were not too slow (<10 s). While the slowing of the ramp strongly decreased the peak current amplitude, the observed charge transfer reduction appeared to be less pronounced.

### Neuronal Activation Is Increased With Intermediately Slow pH Ramps

**Figure 5:**
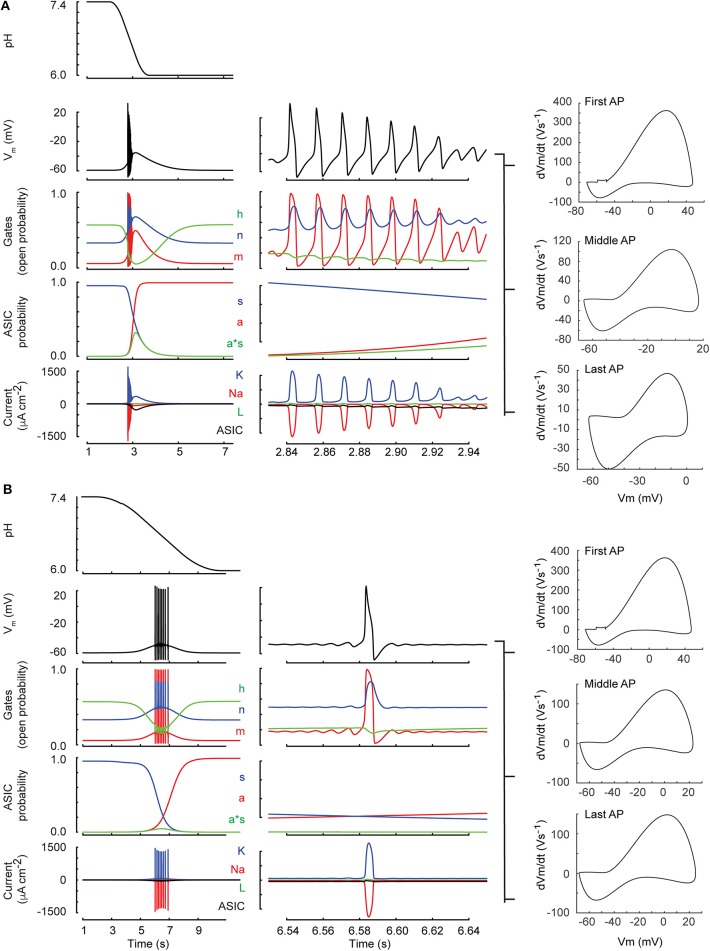
Neuronal model behavior (classical Hodgkin-Huxley model). Shown are two simulations of the neuronal response to a pH drop to pH 6.0 of 1 **(A)** resp. 6 s duration **(B)**. The center column shows a zoom of 120 ms duration selected toward the middle of the firing. From top to bottom, the figures present the time course of the pH, the membrane potential (V_m_), the open probabilities of the gates m, h, and n (see Materials and Methods, section Incorporation of I_ASIC1a_ into the Hodgkin-Huxley Model of the Nerve Action Potential), the ASIC behavior in terms of activation of the activation (a) and the sensitization gate (s), and of the product a·s, and the current related to the voltage-gated sodium (Na) and potassium (K) channels, the leak current (L), and the ASIC-mediated sodium current (ASIC). For both ramp types, phase-plane plots of the first AP, an AP in the middle and the last AP of the burst are shown in the right column.

We investigated then the AP induction by acidification of various speeds. This was first done in a kinetic model, generated by integrating the ASIC1a HH-based model into the classical HH neuronal model (Hodgkin and Huxley, 1952) (Materials and Methods). Figure 5 shows the time course of the simulated pH ramp together with the time course of the membrane potential, the gating variables of the neuron model, the gating variables of the I_ASIC1a_ model as well as the resulting currents in response to a pH 7.4–6.0 ramp of 1 s (Figure 5A) or 5 s duration (Figure 5B). The rapid pH ramp (Figure 5A) caused a rapid activation of I_ASIC1a_ which promptly depolarized the membrane and initiated a burst of APs. However, the burst ended quickly because of prolonged depolarization which inactivated the sodium current (gate *h* dropped to 0). In contrast, with the 5-s ramp (Figure 5B), I_ASIC1a_ was smaller and exhibited a slow time course; thus, the firing occurred later, with a lower frequency and lasted longer because the sodium current was not completely inactivated.

**Figure 6:**
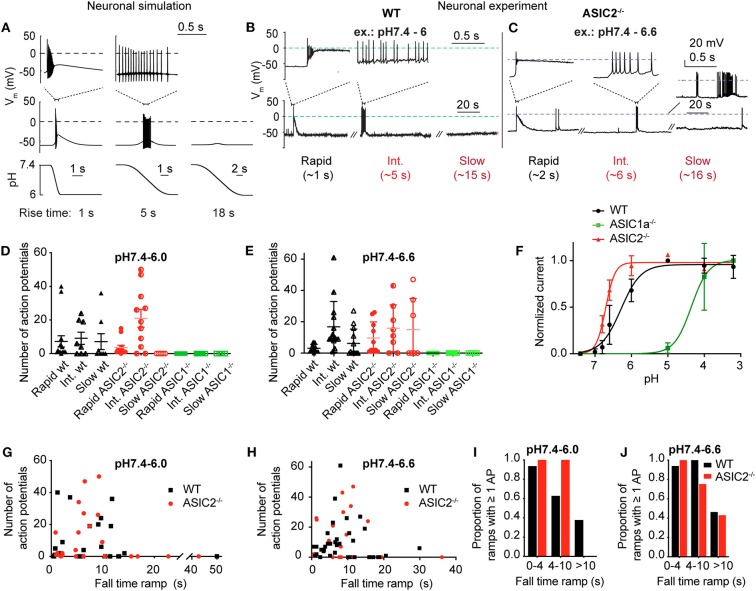
Neuronal modeling and experiments in cortical neurons to assess the response to slow pH changes. **(A)** Simulated neuronal activity in response to pH 7.4–6.0 ramps using the Hodgkin-Huxley (HH) neuronal model in which the ASIC1a model was integrated. Membrane potential changes in response to the pH change (bottom panel) are shown in the middle panel; selected parts of the traces are shown in the top panel on an expanded time scale. **(B,C)** representative experiments with WT (pH 7.4–6.0 ramps) and ASIC2^−/−^ cortical neurons (pH 7.4–6.6 ramps) in current-clamp. Baseline current was injected to obtain a resting membrane potential close to −60 mV. For the slow pH ramp with ASIC2^−/−^ cortical neurons, one trace with practically no (bottom), and one with high activity (top) is shown. **(D,E)** Number of action potentials (APs) induced by rapid (ramp FT < 4 s), intermediate (ramp FT = 4–10 s) and slow (ramp FT > 10 s) pH 7.4–6.0 ramps **(D)** or pH 7.4–6.6 ramps **(E)** of WT, ASIC1a^−/−^ and ASIC2^−/−^ cortical neurons (*n* = 4–15). **(F)** pH dependence of ASIC currents in WT, ASIC1a^−/−^ and ASIC2^−/−^ cortical neurons. Currents were measured in whole-cell patch-clamp at −60 mV. Normalized current amplitudes are plotted, *n* = 2–10. The pH_50_ values were 6.29 ± 0.23 (WT, *n* = 10) and 6.67 ± 0.03 (ASIC2^−/−^, *n* = 4). For ASIC1a^−/−^ neurons, the current amplitudes still increased between pH 4.0 and 3.2, and the pH_50_ is therefore ≤ 4.10 ± 0.04 (*n* = 2). **(G,H)** Number of APs of WT and ASIC2^−/−^ cortical neurons, plotted as a function of the pH ramp FT, in pH 7.4–6.0 ramps (**G**, *n* = 23–28) and pH 7.4–6.6 ramps (**H**, *n* = 23–36). **(I,J)** Proportion of pH 7.4–6.0 **(I)** or pH 7.4–6.6 ramps **(J)** that induced at least 1 AP, presented as probability (*n* = 5–16).

This model predicts that pH 7.4–6.0 pH ramps induce a relatively short burst of APs if the pH change is rapid (ramp FT = 1 s, Figure 6A, left panel), a longer burst if the pH change is intermediately slow (ramp FT = 5 s, center panel), and no AP if the pH ramp is slow (18 s; right panel), because the magnitude of I_ASIC1a_ is then not sufficient to bring the neuronal model to threshold. The validity of the modeling predictions was then tested by measuring AP generation in current-clamp experiments from mouse cortical neurons. pH ramps were applied to cultured cortical neurons of WT, ASIC1a^−/−^ and ASIC2^−/−^ mice. Representative experiments (Figures 6B,C) suggest an increased AP signaling with pH changes of intermediately slow fall time (5–6 s). The quantitative analysis shows first that these pH changes induce APs in WT and ASIC2^−/−^ neurons, but not in ASIC1a^−/−^ neurons (Figures 6D,E). The lack of APs in ASIC1a^−/−^ neurons is due to the shifted pH dependence of ASICs in the absence of ASIC1a (Figure 6F). ASICs of the CNS are mostly ASIC1a homotrimers or heterotrimers composed of ASIC1a, −2a, and/or −2b (Wemmie et al., 2002; Price et al., 2014). The pH_50_ of activation is shifted by ~0.5 units to more alkaline values in ASIC2^−/−^, and by ~2 units to more acidic values in ASIC1a^−/−^ mice (Figure 6F). Previous studies had shown evidence for such differences in pH dependencies between ASIC subtypes, in knockout mice or by recombinant expression of the respective subunit combinations (Baron et al., 2002; Wemmie et al., 2002; Vukicevic and Kellenberger, 2004; Joeres et al., 2016). The experiments with ASIC1a^−/−^ neurons also prove that the acid-induced depolarization that leads to the generation of APs is mediated by the activity of ASICs. Counting of the APs in WT and ASIC2^−/−^ neurons showed that this number does not change significantly with the slowing of the pH ramp in cells in which APs were induced, but that in many experiments, slow pH ramps (FT > 10 s) were not able to induce APs (Figures 6D,E,G,H). While fast pH ramps induced at least one AP in most of the WT and ASIC2^−/−^ neurons, the proportion of pH ramps with ≥1 AP decreased with slower pH ramps (Figures 6I,J).

**Figure 7:**
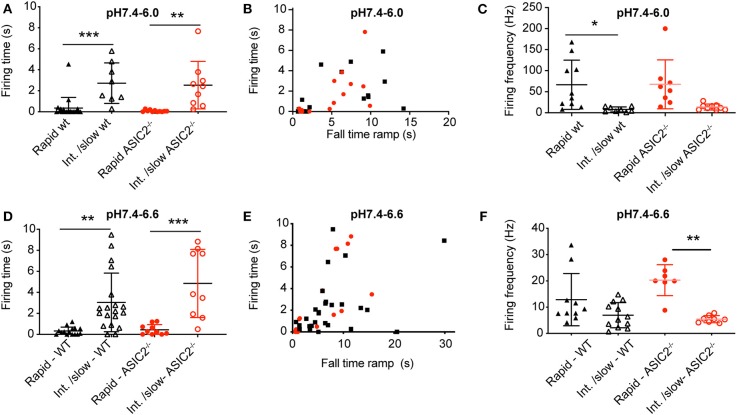
Analysis of acid-induced action potential firing in cultured cortical neurons. The experimental conditions were as described in the legend to Figure 6. **(A,D)** Firing time, calculated as the duration between the beginning of the first and the end of the last AP in AP bursts, plotted for pH 7.4–6.0 ramps **(A)** and pH 7.4–6.6 ramps **(D)** with ramp FT < 4 s (rapid) and >4 s (Int./slow), *n* = 8–20. Only experiments with at least one AP were considered. **(B,E)** Firing time, plotted as a function of the pH ramp FT, for pH 7.4–6.0 ramps **(B)** and pH 7.4–6.6 ramps **(E)**, *n* = 14–22. **(C,F)** AP firing frequency, calculated as AP number/firing time, for pH 7.4–6.0 **(C)** and pH 7.4–6.6 ramps **(F)**, *n* = 7–12. *p < 0.05; **p < 0.01; ***p < 0.001, different between the indicated conditions. Statistical tests were Kruskal-Wallis and Dunn's *post-hoc* test for A and F, ANOVA and Tukey *post-hoc* test for **(C,D)**.

The analysis of the AP burst duration in experiments with induction of at least one AP showed significantly increased bursting times induced by intermediate to slow (FT > 4 s) as compared to fast (FT < 4 s) pH ramps (Figures 7A,B,D,E), in agreement with the model (Figure 6A). The slowing of the pH change lowered the bursting frequency by 2–8-fold in both types of pH ramps tested (Figures 7C,F). Such a slowing of the firing was also predicted by the HH neuronal model, with 60 Hz for a pH 7.4–6.0 ramp with ramp FT of 1 s, and 6.5 Hz for a ramp with a ramp FT of 6 s. This decrease of bursting frequency was due to the lower amplitude of depolarizing I_ASIC1a_.

**Figure 8:**
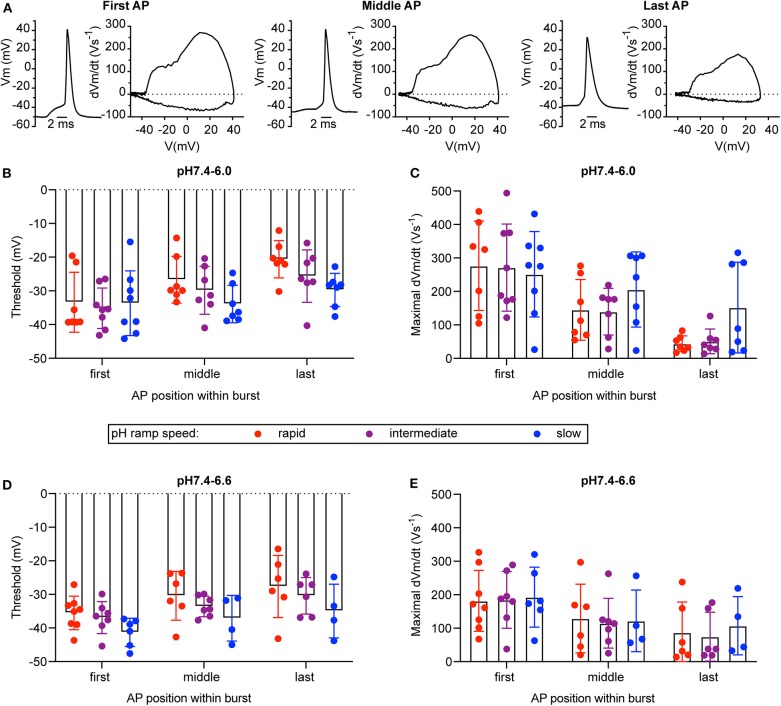
Phase-plane analysis of neuronal action potentials. The data are from current-clamp experiments with WT cultured cortical neurons; baseline current was injected to obtain a resting membrane potential close to −60 mV. The pH ramp protocols produced in control experiments a fall time of 2.0 ± 0.1 s (rapid), 4.4 ± 1.8 s (intermediate), and 17.8 ± 6.7 s (slow; *n* = 3, mean ± SEM), respectively. Of each induced AP burst, the properties of the first, the last, and of an AP in the center of the burst was submitted to the phase-plane analysis. Red, purple, and blue data points were obtained from rapid, intermediate and slow pH ramps, respectively. **(A)** Representative APs and corresponding phase-plane plots of an AP burst induced by a pH 7.4–6.6 ramp of intermediate speed. **(B,D)** Threshold of AP generation in response to pH 7.4–6.0 **(B)** or pH 7.4–6.6 ramps **(D)**, *n* = 4–8. **(C,E)** Maximal AP upstroke velocity measured in experiments with AP induction by pH 7.4–6.0 **(C)** or pH 7.4–6.6 ramps **(E)**, *n* = 4–8. These parameters were determined from phase-plane plots as indicated in Materials and Methods. A two-way ANOVA test identified significant contributions of the AP position to the AP threshold (*p* < 0.001 in pH 7.4–6.0 ramps, *p* < 0.05 in pH 7.4–6.6 ramps) and the maximal AP upstroke velocity (*p* < 0.0001 in pH 7.4–6.0 ramps, *p* < 0.01 in pH 7.4–6.6 ramps) and of the ramp speed to the AP threshold (*p* < 0.05 for pH 7.4–6.0 and pH 7.4–6.6 ramps).

For a series of experiments with WT neurons, a phase-plane analysis (Bean, 2007) was carried out. For these experiments, three different pH ramp protocols were applied, that produced in control experiments a fall time of 2.0 ± 0.1 s (rapid), 4.4 ± 1.8 s (intermediate) and 17.8 ± 6.7 s (slow; mean ± SEM; *n* = 3 per condition), respectively. For each AP burst, a phase-plane analysis, which plots the derivative of the voltage (dV_m_/dt) as a function of the membrane potential V_m_, was made from 3 APs, the first AP, the AP in the middle (in terms of AP counts), and the last AP of the burst. Figure 8A shows representative traces of these 3 APs together with the corresponding phase-plane plots of one burst induced by a pH 7.4–6.6 ramp of intermediate speed. Two parameters that mostly depend on voltage-gated Na^+^ channels were determined from such plots, the spike threshold, identified as the voltage at which dV_m_/dt increases suddenly, and the maximal AP upstroke velocity, corresponding to the maximal value of dV_m_/dt. For both, pH 7.4–6.6 and pH 7.4–6.0 ramps, the analysis of the threshold appeared to be dependent on the order of when the APs occur in time during the pH ramp (first, middle and last AP; *p* < 0.01, two-way ANOVA), with more depolarized threshold for later APs (Figures 8B,D), and on the speed of the pH ramp (*p* < 0.05, two-way ANOVA) with more depolarized threshold measured with fast ramps. The more depolarized threshold of APs occurring at the end of the burst is likely due to an accumulation of inactivated voltage-gated Na^+^ channels (Na_v_s), which results in the fact that a higher membrane depolarization is required for activation of sufficient Na_v_s for the induction of an AP. The higher threshold observed with rapid pH ramps is likely due to the fact that rapid ramps induce strong depolarizations that limit the hyperpolarization between APs. For both, pH 7.4–6.6 and pH 7.4–6.0 ramps, the maximal dV_m_/dt depended on the AP timing (*p* < 0.01, two-way ANOVA), showing highest values for the first AP (Figures 8C,E). The maximal rate of rise of the AP depends mostly on the amplitude of Na^+^ current flowing during the spike (Bean, 2007). Therefore, the maximal dV_m_/dt may be higher in the first as compared to later APs of a burst because during the burst, some Na_v_s do not recover from inactivation and are therefore not available for AP induction.

Together, these observations show that ASIC1a is required for the induction of neuronal activity by acidification to pH 6.6 or 6.0. With pH ramps of FT > 10 s, most of the acidifying ramps did not induce any AP. Intermediate or slow pH ramps (FT > 4 s) that induced APs, generated longer firing times than did fast pH changes. This may be due to the smaller, but longer lasting depolarization induced by intermediate or slow ramps, which allows more complete recovery from inactivation of Na_v_s.

**Figure 9:**
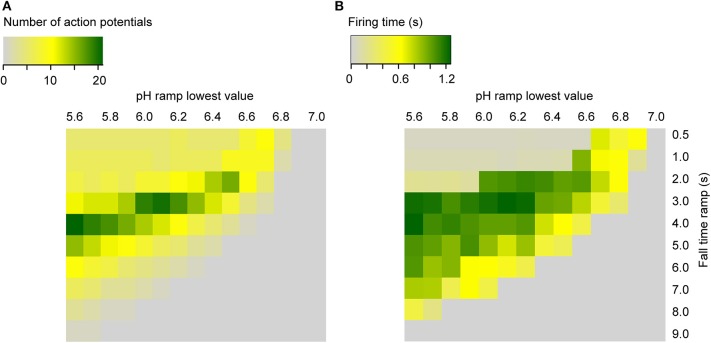
Heat maps of the simulated neuronal activity as a function of the pH ramp lowest value and duration (RT) in seconds, for ramps starting at pH 7.4. Shown are the number of APs **(A)** and the firing time (s) **(B)** for the neuronal Hodgkin-Huxley model integrating ASIC1a.

To predict AP firing for a wider range of pH conditions and ramp speed than was tested experimentally, firing time and AP number were calculated with the kinetic model (using the default parameters, Table 1) for ramps starting at pH 7.4, ramp end pH values of 7.0–5.6, and ramp fall times of 0.5–9 s (Figure 9). This shows that the optimal pH ramp fall time for inducing high numbers of APs and long bursting times is ~3 s at pH 6.0. This optimal pH change speed gets longer with more acidic ramp end pH, and shorter with less acidic values.

### Effects of Variations of Na_v_ Properties on AP Generation in the Neuronal HH Model

**Figure 10:**
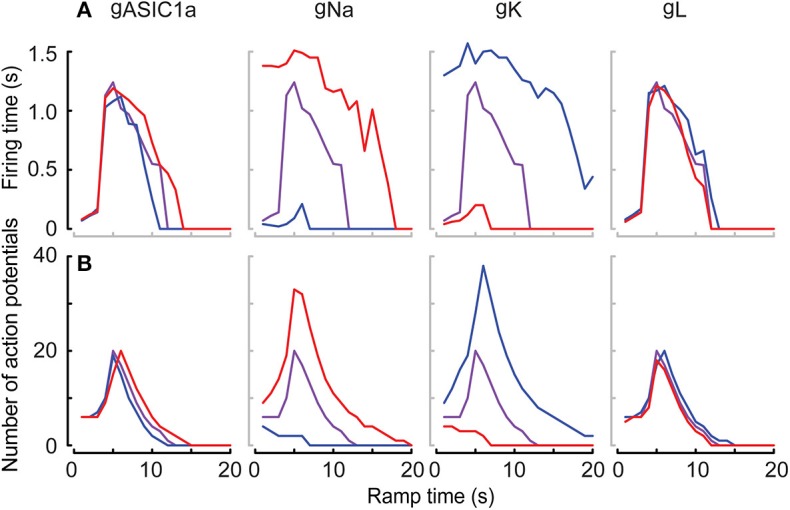
Responses of the neuronal models to the variability of the conductance parameters. Responses in terms of firing time and number of APs of the Hodgkin-Huxley neuronal model integrating the ASIC1a model with pH 7.4–6.0 ramps. The differently colored lines highlight the effect of varying the maximal conductance of ASIC1a (g_ASIC_), sodium (g_Na_), and potassium (g_K_) channels, as well as of the leak current (g_L_) by ±10% of the optimized corresponding parameters. The firing time **(A)** and the number of APs **(B)** are shown in purple for optimized parameters, and as blue and red lines for optimized corresponding parameters −10% (blue) and +10% (red). Note that the speed of pH ramps is presented here as total ramp time, and not ramp FT, as in other figures.

The high neuron-to-neuron variability with regard to AP numbers and firing time is likely due to differences in expression levels of ASICs, but also of other ion channels involved in AP generation in these neurons. To determine in the kinetic model how the AP number and firing time depend on the amplitude of different currents, the maximal conductance of the three currents in the neuronal HH model (Na^+^ current, K^+^ current and leak current) and the maximal conductance of the ASIC1a current were individually increased or decreased by 10%, and AP numbers and firing time were determined in pH 7.4–6.0 ramps. This analysis showed that the magnitude of the Na^+^ and K^+^ currents (given by their respective conductances g_Na_ and g_K_) exert a strong influence on AP number and firing time (Figure 10).

**Figure 11:**
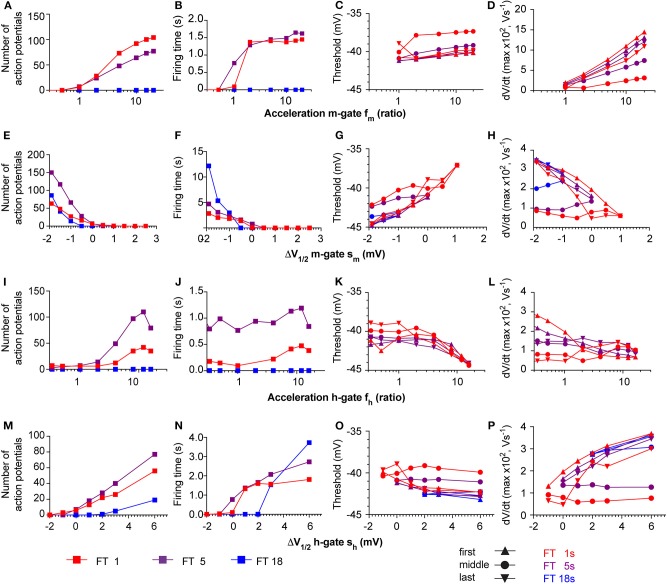
Responses of the neuronal models to variations of Na_v_ parameters. The neuronal modeling results are summarized for pH ramps of three durations [short, FT (=fall time) 1 s; intermediate (FT 5 s); slow (FT 18 s)], and in which the following Na_v_ parameters were varied: Kinetics of the m-gate, f_m_ **(A–D)**, shift of the V_1/2_ of the m-gate, s_m_ **(E–H)**, kinetics of the h-gate, f_h_ **(I–L)**, and shift of the V_1/2_ of the h-gate, s_h_ **(M–P)**. The responses are shown in terms of number of APs (1st column from left), firing time (2nd column from left), and two parameters from phase-plane plot analysis of the first, middle and last AP in the burst, the spike threshold (3rd column from left) and the maximal AP upstroke velocity (1st column from right). The colors of the symbols refer to the speed of the pH ramp, and the symbol types in the two right columns refer to the position of the studied AP within the burst, as indicated in the figure.

The neuronal kinetic model used in this study includes besides the ASIC conductance one Na_v_ and one K_v_ conductance, plus a leak conductance. In individual mammalian central neurons, 2–3 components of Na_v_ current and 4–5 different components of K_v_ currents are found, besides other current types that are not part of the neuronal model used here (Bean, 2007). Nine different Na_v_ subtypes are known, which have distinct kinetics and voltage dependence. Especially some of the Na_v_ subtypes expressed in sensory neurons of the peripheral nervous system deviate in their voltage dependence and kinetics strongly from other Na_v_s (Bennett et al., 2019). To determine how such differences in Na_v_ current properties affect AP induction, a series of additional simulations with the HH-based neuronal model was carried out, in which the kinetics and voltage dependence of the activation (m) and the inactivation (h) gate of the Na_v_ current were systematically varied. To model changed kinetics, forward and backward rates were scaled by the same factor f_m_ or f_h_, in order not to affect the equilibrium constant at the same time (i.e., the potential of half steady state activation/inactivation V_1/2_ remains the same). In this modified model, pH ramps of three different speeds (fall time of 1, 5, or 18 s, corresponding to a rapid, intermediate and slow pH ramp) were applied, and in each condition, the number of APs, the firing time as well as two parameters measured from phase-plane plots (Figure 5, right column), the AP threshold and maximal dV_m_/dt were determined (Figure 11). With the default parameters of the model (as described in Table 1 and illustrated in Figures 5, 6A), the V_1/2_ of activation and inactivation are −21.6 and −57.3 mV, respectively, and the rates of activation and inactivation at 0 mV are 3.8 and 0.1 ms^−1^, respectively, and the model induced APs with the fast and the intermediate, but not with the slow pH 7.4–6.0 ramp (Figure 6A). In these conditions, the parameters derived from the phase-plane plot depended on the timing of the AP in the burst: the threshold was slightly less negative for APs occurring later in the burst, and the maximal AP upstroke velocity was decreased (compare values at f_m_ = 1 or s_m_ = 0 in Figures 11C,D,G,H, respectively), reproducing therefore the observations made in the cortical neurons (Figure 8).

Slowing of the kinetics of the m-gate reduced the number of APs and the firing time, while acceleration of these kinetics increased, in a saturating way, these two parameters (Figures 11A,B). The AP threshold remained within relatively narrow limits (Figure 11C), while the maximal slope increased when the activation was accelerated (Figure 11D), as expected if more Na_v_ channels open in the same unit of time.

The model proved to be extremely sensitive to changes in the voltage dependence of the m-gate. Negative shifts s_m_ of the m-gate V_1/2_ of < −2.5 mV induced spontaneous AP signaling, while small positive s_m_ (e.g., +2 mV in 1 s pH ramps) precluded any AP induction. A negative s_m_ allowed also slow pH ramps (FT of 18 s) to induce AP bursts (Figures 11E,F). It is expected that larger positive s_m_ would be tolerated if gNa was increased at the same time. However, because increases of gNa by >10% induced spontaneous AP signaling, the gNa was not changed in these simulations. Negative s_m_ increased as expected the number of APs and the burst duration (Figures 11E,F). The AP threshold remained within narrow limits and showed a slight depolarizing shift when V_1/2_ was increased (Figure 11G), while the maximal AP upstroke velocity decreased (Figure 11H). In this series of simulations, the APs in the middle of the burst had generally a higher AP threshold and a lower maximal AP upstroke velocity than the first and the last AP of the same burst. This difference is due to the fact that in the middle of the AP burst, the ASIC-induced depolarization is of higher amplitude than at the beginning and the end of the ramp, and limits the recovery from inactivation of Na_v_s, allowing less Na_v_s to open at the same time.

Acceleration of the kinetics of the h gate (f_h_ > 1) increased generally the number of APs and to a lesser extent the burst duration (Figures 11I,J). The acceleration of the h gate speeds up not only inactivation, but also the recovery from inactivation. For this reason, more APs were induced with the same ASIC-mediated depolarization. The AP threshold became slightly more negative with accelerated kinetics of the h gate (Figure 11K), and a decrease was observed for the maximal AP upstroke velocity of the first AP (Figure 11L). This is likely due to a reduction of overlapping openings, due to the accelerated inactivation. In contrast, the maximal slope of the middle and last AP did practically not depend on the h gate kinetics.

Small negative shifts of the h gate V_1/2_ (s_h_ < −1 mV) prevented AP induction. Positive shifts increased the number of APs and the firing time, and allowed also AP induction with slow pH ramps (FT = 18 s; Figures 11M,N). The AP threshold underwent a small negative shift with more positive s_h_ (Figure 11O), and the maximal dV_m_/dt increased at the same time (Figure 11P). The increased maximal dV_m_/dt paralleling a positive s_h_ is caused by a more complete recovery from inactivation of the Na^+^ current at the onset of the AP. However, similar to the simulations in which s_m_ was varied, the APs in the middle of the burst showed a divergent behavior compared to the first and the last AP. Together, these simulations show how changes in Na_v_ properties, which could stand for an exchange of the expressed Na_v_ subtype, affect ASIC-mediated AP induction.

## Discussion

We provide here a quantitative description of the dependence of ASIC1a-mediated currents and charge transport during solution changes taking ~1–40 s to develop, and of the dependence of AP firing in cortical neurons on the amplitude and speed of the pH change. We show that slowing of the pH change reduced both ASIC1a peak currents and the ASIC1a-mediated charge transport. In mouse cortical neurons, ASICs can induce APs in response to acidification. ASIC activation induced longer firing times compared to fast changes if the pH change was intermediately slow (FT = 4–10 s). Slow pH changes (FT > 10 s) induced however only rarely AP bursts.

To obtain deeper insight into the interaction between acidification kinetics and I_ASIC1a_, we developed a model of I_ASIC1a_ based on the Hodgkin-Huxley formalism. While many Markovian models of ASIC function have been published (Li et al., 2010, 2012; Blanchard and Kellenberger, 2011; Grunder and Pusch, 2015; Vullo et al., 2017), their adjustment to other experimental conditions and the proper identification of their parameters is not always straightforward (Fink and Noble, 2009). Therefore, we opted for the simpler, but more robust approach of Hodgkin and Huxley, and we observed that such a model is sufficient to describe our most salient experimental observations. When applied to pH ramps, the kinetic ASIC model fitted the peak current amplitudes well, while the charge transport in pH 7.4–6.6 ramps was less faithfully reproduced. There was also some discrepancy in the current kinetics induced by pH ramps. The kinetics of current appearance were slower, and the peak appeared later in the simulated traces when compared to the representative measured current traces (Figure 4B). Since the kinetics of ASIC1a currents induced by rapid pH changes were faithfully reproduced by the kinetic model (Figure 2B), these small, but clear differences highlight the limits of the model in the specific context of the slow pH changes, which may be due to the design of the model itself, or sub-optimal parameters in this pH range. This discrepancy may also have arisen from the impossibility to replicate in the simulations the exact time course of pH in the experiments.

We have recently found that the human ASIC1a clone used as wild type by many laboratories [GenBank accession number U78181 (Garcia-Anoveros et al., 1997)] carries the mutation G212D (Vaithia et al., 2019). The experiments with recombinant human ASIC1a in the present study were carried out with the ASIC1a clone containing the G212D mutation. The main effect of the G212D mutation is an acceleration of the current decay kinetics [τ_pH6_ = 0.35 ± 0.06 s, *n* = 8 (ASIC1a-212D) vs. 1.25 ± 0.20 s, *n* = 7 (ASIC1a-212G)] (Vaithia et al., 2019). Besides, the SSD occurs at slightly more alkaline pH in ASIC1a-212D (alkaline shift of 0.08 pH units of the midpoint of SSD for ASIC1a-212D compared to ASIC1a-212G) (Vaithia et al., 2019). The sustained/peak current ratio is however not affected (Vaithia et al., 2019). Our experiments with recombinantly expressed ASIC1a, and the kinetic model based on them may therefore somewhat underestimate the contribution of ASIC1a. The relatively close agreement between the neuronal modeling results and the AP induction properties in mouse cortical neurons suggest that these differences are small.

While ASIC1a current amplitude and charge transport decreased in parallel with the speed and amplitude of the pH change, we observed that a slight slowing of the pH change induced in fact longer AP bursts. Fast changes to pH 6.0 induced in many experiments very strong depolarizations with only a short burst of AP firing. We have previously shown that strong acidification-induced depolarization can reduce AP firing (Vukicevic and Kellenberger, 2004), probably due to inactivation of voltage-gated Na^+^ channels. Slower developing acidifications (FT 4–10 s) depolarized the membrane potential less, but over a longer time, inducing more efficiently long bursts of APs. Slow pH changes (FT > 10 s) induced only rarely APs. This biphasic dependence on the speed and amplitude of the pH change was reproduced by the neuronal model, allowing us to expand the predicted ASIC response beyond the conditions tested experimentally. By varying the conductance of the different ionic current types in the neuronal model and calculating the effects of these changes on AP numbers and firing time, we could show that these two characteristics depend not so much on the ASIC current density, but more on the conductance density of voltage-gated Na^+^ and K^+^ channels involved in AP generation (Figure 8).

To estimate how the expression of different Na_v_ subtypes with distinct properties may affect acid-induced AP induction, the gating parameters of the Na_v_ current in the neuronal model were systematically varied. Although this analysis revealed a higher sensitivity of the model to parameter changes than the expected sensitivity of real neurons, it provided useful information on the influence of Na_v_ properties on AP generation. The analysis showed that acceleration of the kinetics of the m-gate and a negative shift of its voltage dependence both increased the number of APs and the firing time. Concerning the h gate, acceleration of the kinetics and a positive shift of its voltage dependence increased the number of action potentials and the firing time. This indicates that for example an acceleration of the kinetics of recovery from inactivation that is observed with Nav1.3 and Nav1.8 (Bennett et al., 2019) would increase the number of APs relative to the default parameters of the HH neuronal model. The model also suggested that changes of the kinetics alone, without shifts in the voltage dependence, would not allow AP generation with the slow pH ramps of 18 s FT.

Our neuronal model predicts AP signaling based on the ASIC properties, on the conductance of Na^+^, K^+^ and leak currents, as well as on the voltage dependence and kinetics of Na^+^ channels. An earlier study has shown that ASIC1a can interact with big conductance Ca^2+^-activated K^+^ (BK) channels (Petroff et al., 2012). This interaction inhibits the BK K^+^ currents, and the inhibition is partially removed by acidification; thus, acidification would increase the BK currents. BK channels can have different functions, depending on the context, and it was shown in several studies that increased BK currents result in a faster recovery of Na^+^ channels from inactivation and increase therefore neuronal excitability (Kshatri et al., 2018). BK current densities and neuronal excitability were indeed higher in neurons of ASIC1a/2/3^−/−^ mice (Petroff et al., 2012). The removal of ASIC-mediated BK channel inhibition may therefore, in addition to the depolarization-mediated activation of Na_v_s, contribute to some extent to the increased excitability induced by an acidification.

Slow pH changes in the nervous system were reported to have kinetics corresponding to ramp fall times longer than 10 s (Kraig et al., 1983; Chesler, 2003), the value that our experiments suggest as an upper limit for pH changes still efficiently inducing APs. ASICs have however well-documented roles in settings associated with such slow pH changes, as e.g., ischemia (Xiong et al., 2004) and seizures (Ziemann et al., 2008; Lv et al., 2011). A recent study has shown that in ischemia-like conditions, ASIC1a activation can induce necroptosis by a mechanism that is independent of ion conductance by ASIC1a (Wang et al., 2015), and may therefore be operational with slowly developing acidification. Regarding other roles of ASICs linked to slow pH changes, it is important to note that in recent years, several molecules besides protons, such as e.g., some specific lipids have been shown to activate or potentiate ASICs (Smith et al., 2007; Li et al., 2011; Marra et al., 2016). Some of these molecules are endogenous mediators, such as the lipids arachidonic acid and lysophosphatidylcholine (Smith et al., 2007; Marra et al., 2016) or the gasotransmitter nitric oxide (Cadiou et al., 2007). These, or related, currently unknown mediators may allow sufficient ASIC activation by slowly developing pH changes. The study of the activity changes of endogenous mediators during processes such as inflammation or ischemia may help identifying such ASIC modulators.

In conclusion, ASIC function has so far only been measured in response to fast pH changes, although it is known that several processes, for which a contribution of ASICs is firmly established, involve slowly developing and long-lasting pH changes. We provide here the first analysis of ASIC1a responses to defined slow pH changes. We show that moderate slowing of the acidification time course (4–10 s) can, although reducing the ASIC1a current amplitudes, lead to an increase in AP signaling in brain neurons. Slower acidification (>10 s) was inefficient in inducing ASIC currents and AP signaling in our experimental conditions. It is likely that modulatory mechanisms that are currently incompletely understood allow sufficient ASIC activity in such situations.

## Data Availability Statement

All datasets generated for this study are included in the article/Supplementary Material.

## Ethics Statement

The animal study was reviewed and approved by Committee on animal experimentation of the Canton de Vaud.

## Author Contributions

The project was designed by OA, SK, and JK. OA and ZP carried out the experiments. EH, OB, and JK carried out the computational work. SK, OA, and JK wrote the manuscript. All authors approved the final manuscript.

### Conflict of Interest

The authors declare that the research was conducted in the absence of any commercial or financial relationships that could be construed as a potential conflict of interest.

## Abbreviations

AP: action potential
ASIC: acid-sensing ion channel
BK channel: big conductance Ca^2+^-activated K^+^ channel
CHO: Chinese hamster ovary
COD model, triangular kinetic ASIC model based on a closed: an open and a desensitized state
FBS: fetal bovine serum
FCS: fetal calf serum
FT: fall time = time to pass from 10 to 90% of the maximal change in signal amplitude
HH: Hodgkin-Huxley
Na_v_: voltage-gated Na^+^ channel
pH_50_: pH of half-maximal activation
SSD: steady-state desensitization
WT: wild-type.
